# Genetic and Clinical Analyses of DOA and LHON in 304 Chinese Patients with Suspected Childhood-Onset Hereditary Optic Neuropathy

**DOI:** 10.1371/journal.pone.0170090

**Published:** 2017-01-12

**Authors:** Yadi Li, Jie Li, Xiaoyun Jia, Xueshan Xiao, Shiqiang Li, Xiangming Guo

**Affiliations:** State Key Laboratory of Ophthalmology, Zhongshan Ophthalmic Center, Sun Yat-sen University, Guangzhou, China; National Eye Institute, UNITED STATES

## Abstract

Leber hereditary optic neuropathy (LHON) and dominant optic atrophy (DOA), the most common forms of hereditary optic neuropathy, are easily confused, and it is difficult to distinguish one from the other in the clinic, especially in young children. The present study was designed to survey the mutation spectrum of common pathogenic genes (*OPA1*, *OPA3* and mtDNA genes) and to analyze the genotype-phenotype characteristics of Chinese patients with suspected childhood-onset hereditary optic neuropathy. Genomic DNA and clinical data were collected from 304 unrelated Chinese probands with suspected hereditary optic neuropathy with an age of onset below 14 years. Sanger sequencing was used to screen variants in the coding and adjacent regions of *OPA1*, *OPA3* and the three primary LHON-related mutation sites in mitochondrial DNA (mtDNA) (m.3460G>A, m.11778G>A and m.14484T>C). All patients underwent a complete ophthalmic examination and were compared with age-matched controls. We identified 89/304 (29.3%) primary mtDNA mutations related to LHON in 304 probands, including 76 mutations at m.11778 (76/89, 85.4% of all mtDNA mutations), four at m.3460 (4/89, 4.5%) and nine at m.14484 (9/89, 10.1%). This result was similar to the mutation frequency among Chinese patients with LHON of any age. Screening of *OPA1* revealed 23 pathogenic variants, including 11 novel and 12 known pathogenic mutations. This study expanded the *OPA1* mutation spectrum, and our results showed that *OPA1* mutation is another common cause of childhood-onset hereditary optic neuropathy in Chinese pediatric patients, especially those with disease onset during preschool age.

## Introduction

Hereditary optic neuropathies are a group of diseases that have highly heterogeneous genetic and clinical characteristics and a minimum prevalence of one in 10000 [1]. These diseases include Leber hereditary optic neuropathy (LHON, OMIM 535000) and dominant optic atrophy (DOA, OMIM 165500), which are primarily caused by mutations in mitochondrial DNA (mtDNA) and nuclear genes, respectively. Both LHON and DOA are associated with damage restricted to the retinal ganglion cells and their axons caused by mitochondrial dysfunctions and characteristically manifest as painless, bilateral vision loss and symmetrical temporal pallor of the optic nerve [2–4].

DOA was first described by Kjer and is classically understood as an isolated optic neuropathy [5,6]. Although many classes of genes have been associated with DOA, extensive reports have primarily correlated DOA with genetic mutations in *OPA1* (OMIM 605290) and *OPA3* (OMIM 606580), which are responsible for the regulation of mitochondrial morphology [7,3,8,9]. Mutations in *OPA1* accounted for approximately 60–80% of all cases of DOA [5,10,11]. Clinically, DOA of childhood onset manifests as insidious, bilateral vision loss with best-corrected visual acuity ranging from 0.15 to 0.5 [12,13]. DOA patients with *OPA1* mutations present with symmetrical temporal optic atrophy or diffused optic nerve pallor on ophthalmic examinations, along with diffuse or temporal thinning of the optic nerve fibers on optical coherence tomography (OCT) [12,14,15]. LHON, which was initially described by Theodor Leber, is typically a maternally inherited optic neuropathy; more than 90% of all LHON patients harbour one of three primary mtDNA mutations (m.11778G>A, m.14484T>C, and m.3460G>A) [16–18]. LHON generally affects young males, often manifesting as synchronous or sequential acute or subacute vision loss in one eye, with the second eye involved within one year [19–21]. In the acute stage of LHON (LHON-A), patients present with telangiectatic vessels surrounding the optic discs and edema in the retinal nerve fiber layer (RNFL) [22,23]. At six months to one year after disease onset, LHON slowly progresses to a static phase in which patients exhibit painless central vision loss and optic disc atrophy (LHON-SP) [24,25]. There is an overlap between *OPA1* mutations and LHON, and LHON progresses to an end stage characterized by bilateral optic atrophy and central vision loss [26–28].

Recently, many studies analyzed the genes associated with suspected hereditary optic neuropathy in Chinese populations of any age [29,30], however disease onset in childhood was reported as rare. Although DOA commonly initially manifests in patients during childhood while LHON affects juveniles and youths [6,19], an Italian study reported that childhood onset of LHON accounted for 11.5% of cases among pediatric patients with hereditary optic neuropathy onset below 10 years [31]. Patients presenting with LHON in childhood are more likely to skip the acute stage and directly progress to optic nerve atrophy, which is more difficult to distinguish from DOA [26–28]. Nevertheless, DOA and LHON patients with earlier onset had better vision and prognosis than those with adult onset [31–33,6]. With increasing age, both DOA and LHON patients can experience additional symptoms [34–37,28]. Therefore, the sooner the disease is diagnosed, the sooner the entire family can receive accurate genetic counseling and avoid the subsequent harm caused by DOA, LHON, and their associated syndromes [35,34,36]. In this cohort study, we screened the common gene mutation spectrum of hereditary optic neuropathy (*OPA1*, *OPA3* and the three primary LHON-related mtDNA mutation sites) and analyzed the clinical characteristics of Chinese patients with suspected hereditary optic neuropathy with an age of onset no more than 14 years.

## Methods

### Patients

Data from 304 unrelated probands with childhood onset (no more than 14 years of age) of suspected hereditary optic neuropathy in the Pediatric and Genetic Clinic of Zhongshan Ophthalmic Center were collected from January 2011 to December 2015. All the probands were assessed by experienced ophthalmologists and were recruited based on the following criteria: (1) variable degree of bilateral optic edema, hyperaemia or pallor on a fundus examination; (2) impaired visual acuity without other ocular diseases such as ametropia, ocular media opacity or retinal diseases; and (3) no optic atrophy caused by tumour, trauma, inflammation or glaucoma.

This study was conducted with the approval of the Institutional Review Board of Zhongshan Ophthalmic Center, Sun Yat-Sen University. Before recruitment into our study, all participants and their guardians signed written informed consent in accordance with the guidelines of the Declaration of Helsinki. This research abided by the process of collection of data from patients with genetic diseases and the requirements of the Ministry of Public Health of China.

### Mutation screening

#### Screening strategy

We first screened for the three primary mtDNA mutations (m.11778G>A, m.14484T>C m.3460G>A) in all 304 patients and then screened for mutations in *OPA1* and *OPA3* in the patients without mtDNA mutations utilizing the ABI BigDye Terminator cycle sequencing kit v3.1 and an ABI 3100 Genetic Analyzer (Applied Biosystems, Foster City, CA). All of the primers for *OPA1* and *OPA3* amplification and sequencing are listed in the S2 and S3 Tables.

### Analysis of mutations

Sequence alignment was conducted using SeqMan II software (Lasergene8.0; DNAStar, Madison, WI). Amino acid changes resulting from missense variants were predicted using Polyphen-2 (PPH2, http://genetics.bwh.harvard.edu/pph2/) and sift (http://sift.jcvi.org). The mutant frequency was acquired from EVS (Exome Variant Server, http://evs.gs.washington.edu/EVS/) and the 1000 Genomes Project database (http://browser;1000genomes.org/). Splicing site variants within noncoding regions and anonymous variations were evaluated using BDGP (http://www.fruitfly.Org/seq_tools/splice.html). The conservation of amino acids variants was analyzed with the MegAlign program in the Lasergene package. All the variants were confirmed by bi-directional sequencing. All mutations were verified in 96 normal controls, as well as in the available family members.

### Clinical data collection and statistical analysis

All the probands received a comprehensive ophthalmic examination, including a best-corrected visual acuity assessment, ophthalmoscopic observation and imaging, OCT and visual evoked potential (VEP) measurement. All of the statistical analyses were performed using SPSS software version 13.1. The age of disease onset and visual acuity in probands with OPA1 mutations and LHON (LHON-A or LHON-SP) were compared using independent-samples t test. The RNFL thickness was compared among DOA probands with *OPA1* mutations and patients with LHON and controls via one-way analysis of variance (ANOVA). The comparison of proband visual acuity between the missense and other mutation types in the *OPA1* gene was conducted using independent-samples t test. The subjects in the control group for comparison of RNFL parameters included healthy children that visited our hospital for physical examinations at an age of no more than 14 years.

## Results

Three hundred and four probands with suspected childhood-onset hereditary optic neuropathy were examined in this study, including 69 females and 235 males. The onset age of the probands ranged from 1–14 years (8.7 ± 3.4 years; mean ± SD) years, and 20.1% (61/304) had a family history of hereditary optic neuropathy. Molecular defects were identified in 119/304 (39.1%) probands; 89/119 (74.8%) of these individuals carried one of the three primary mtDNA mutations (LHON group), 26/119 (21.8%) carried an *OPA1* mutation (the DOA group consisted of those with an *OPA1* mutation), and 4/119 (3.4%) carried an *OPA3* mutation (the DOA group consisted of those with an *OPA1* or *OPA3* mutation). In probands with onset at preschool age (not more than six years), the frequency of *OPA1* mutations (22.3%) was clearly higher than that of LHON-related mutations (6.4%) (Table 1). For probands with an onset age of 7–14 years, the frequency of *OPA1* mutations (2.4%) was clearly lower than that of the three primary mtDNA mutations (39.5%) (Table 1).

**Table 1:** The mutational spectrum of probands harbouring mtDNA and *OPA1* mutations.

Mutation	Below 14 years	Below 10 years	Below 6 years	7–14 years	F / S	F / M	Onset age
**Total**	119/304	63/201	29/94	90/210	61/243	69/235	8.7 ± 3.4
**mtDNA**	89/304	38/201	6/94	83/210	30/59	10/79	10.6 ± 2.8
**OPA1**	26/304	24/201	21/94	5/210	8/18	4/22	5.8 ± 3.4
**OPA3**	4/304	1/201	2/94	2/210	2/2	0/4	6 ± 3.16

F / S: Familial / Sporadic; F / M: Female / Male

### mtDNA mutations and clinical characteristics

**Fig 1:**
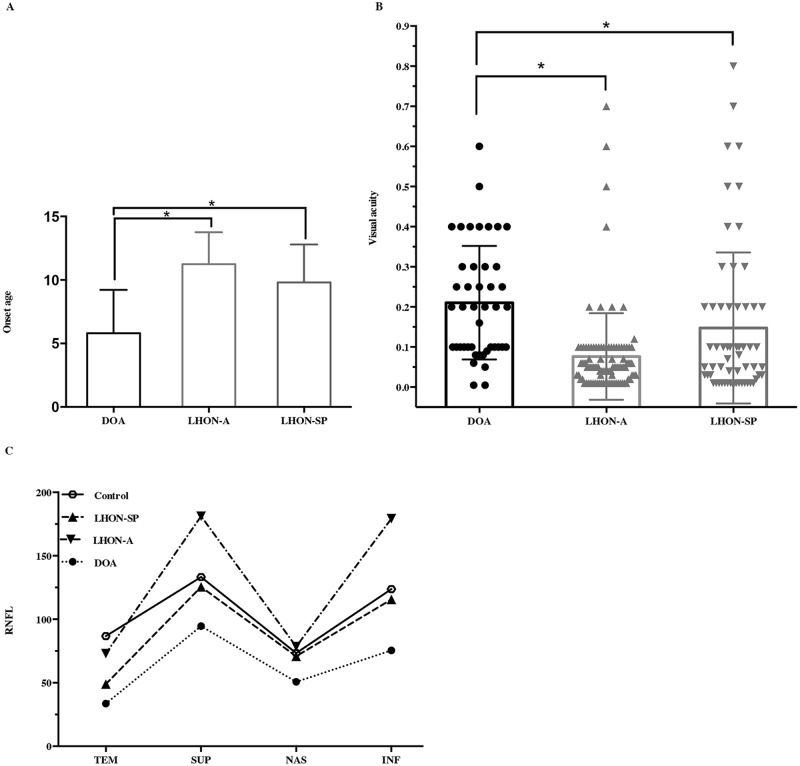
Comparison of the onset age, visual acuity and RNFL thickness in the DOA and LHON (LHON-A and LHON-SP) groups. (A) Comparison of the onset ages of the DOA, LHON-A and LHON-SP: (a) DOA at preschool age; (b) LHON-A and LHON-SP groups at school age; (c) the onset age of DOA was statistically younger than LHON-A (P < 0.001) and LHON-SP (P < 0.001). * The onset age of DOA was earlier than patients with LHON-A (P < 0.001) and LHON-SP (P < 0.001). (B) Comparison of the visual acuity among DOA, LHON-A and LHON-SP: (a) The visual acuity of LHON-A was worse than LHON-SP; (b) The visual acuity of DOA was significantly better than LHON-A and LHON-SP. * The visual acuity of patients with OPA1 mutations was significantly better than patients with LHON-A and LHON-SP (P < 0.001). (C) Comparison of RNFL thickness in four quadrants among patients with OPA1 mutations, LHON-A, LHON-SP and controls using one-way ANOVA: (a) The RNFL was thinner in all four quadrants of DOA. (b) The RNFL thickness of LHON-A group was statistically thicker in superior and inferior quadrants, but there were no obvious differences in the temporal and nasal quadrants. (c) The RNFL thickness of LHON-SP was significantly thinner in the temporal quadrant, but there were no significant differences in the other three quadrants. DOA: patients with *OPA1* mutations; LHON-A: patients with LHON presented with optic edema or hypaeremia with tortuous vessels. LHON-SP: patients with LHON presented with optic pallor or atrophy.

**Fig 2:**
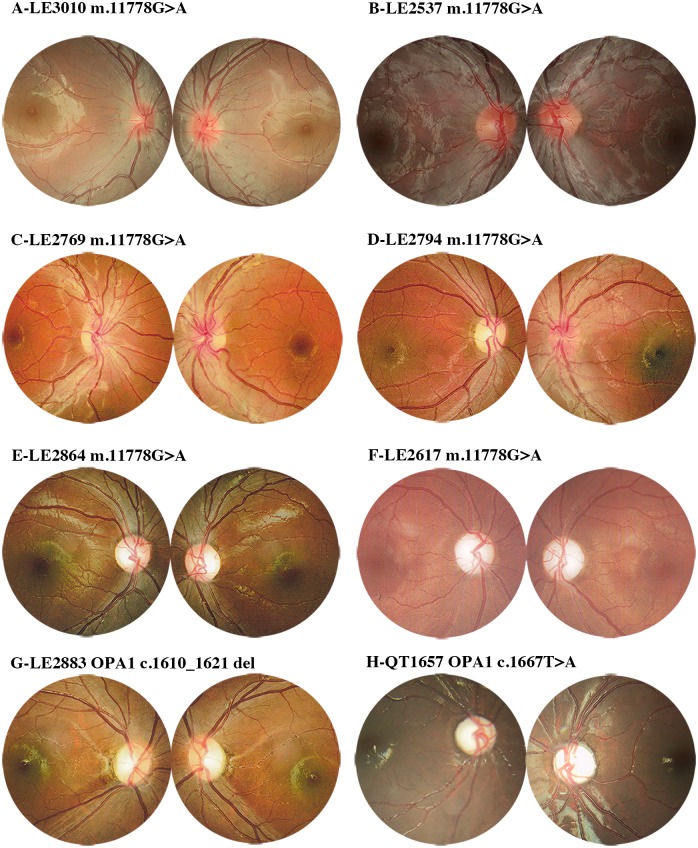
Fundus manifestations of patients with DOA and LHON. (A-F) LHON-A (edema of optic nerve) patients manifested as: (A) bilateral optic nerve edema without telangiectatic vessels; (B) bilateral optic nerve hyperaemia with telangiectatic vessels and microvascular tortuosity (C) temporal optic pallor with vascular tortuosity in both eyes; (D) temporal optic pallor with microvascular tortuosity in the right eye and optic nerve hyperaemia with telangiectatic vessels in left eye. LHON-SP manifested as: (E) temporal optic atrophy without changes in the vessels in both eyes; (F) diffused atrophy of the optic disk in both eyes. (G-H) The manifestations of DOA (G-H) were similar to LHON-SP with temporal optic atrophy (E, G) or diffused optic pallor (F, H). The patients IDs and mutations are located above the fundus images.

Based on the sequencing results, 89 of the 304 probands (29.3%) carried one of the three primary mtDNA mutations (LHON group). Among this group, mutations at m.11778, m.14484 and m.3460 were observed in 85.4% (76/89), 10.1% (9/89) and 4.5% (4/89) of the cases, respectively. Additionally, the LHON group included 79 males and 10 females, and one-third (30/89) had a family history of LHON. Furthermore, 54 of the 89 probands had optic edema and tortuous vessels (acute stage of LHON, LHON-A) in at least one eye, and the remaining 35 probands had binocular optic pallor or atrophy (slowly progressive stage of LHON with optic pallor, LHON-SP). The average age of LHON onset was nearly 10 years (10.6 ± 2.8 years; mean ± SD). The age of disease onset in patients with LHON-SP (9.8 ± 3.0 years; mean ± SD) was significantly earlier than patients with LHON-A (11.1 ± 2.5 years; mean ± SD, P < 0.05) (Fig 1A). The mean visual acuity of the patients with LHON was 0.11 ± 0.15 (mean ± SD). The visual acuity of patients with LHON-A (0.06 ± 0.46, mean ± SD) was worse than those with LHON-SP (0.12 ± 0.03, mean ± SD; P < 0.001). Fundus manifestations of LHON are shown in Fig 2. The patients with LHON-A exhibited optic edema with (Fig 2B) or without (Fig 2A) telangiectatic vessels, and the patients with LHON-SP exhibited binocular temporal (Fig 2E) or diffuse optic atrophy (Fig 2F). Compared with age-matched control children, the 13 patients with LHON-A showed a significantly greater RNFL thickness (as measured by OCT) in the superior (181.15 ± 34.49 μm, P < 0.001) and inferior (179.35 ± 29.26 μm, P < 0.001) quadrants, but no evident differences in RNFL thickness were observed in the temporal (73.04 ± 27.17 μm, P = 0.656) and nasal (70.80 ±11.99 μm, P = 1.0) quadrants. For the group of five patients with LHON-SP compared to the control group, the RNFL thickness was significantly reduced in the temporal (49.00 ± 13.72 μm, P = 0.001) quadrant, while no differences in RNFL thickness were observed in the other three quadrants (superior quadrant, 125.50 ± 21.75 μm, P = 0.977; nasal quadrant, 70.80 ± 11.99 μm, P = 0.994; and inferior quadrant, 115.6 ± 33.27 μm, P = 1.000) (Fig 1C). The VEP pattern in the LHON patients ranged from a serious conduction defect to the absence of a waveform.

### OPA1 mutations and clinical characteristics

Based on screening of the entire coding exons and adjacent introns of *OPA1*, 23 different pathogenic mutations, including 12 previously reported and 11 novel mutations (S1 Fig) were detected in 26 (8.6%) of the 304 probands (Table 1). All the mutations were predicted to be pathologic according to bio informative analysis. The 23 mutations included 10 (43.5%) missense, seven (30.4%) splicing site, three (13.0%) frame-shift, two nonsense and one in-del mutations. Seven (26.9%, 7/26) of these probands harboured *OPA1* mutations located in exon 12, and four (15.4%, 4/26) in exon 27 (Table 2). All of the mutations were absent from the 96 normal controls alleles. Twenty-two males and four females carried *OPA1* mutations, and eight (8/26, 30.8%) patients had a family history of DOA. The age of onset of the DOA patients ranged from 1 to 14 years (5.8 ± 3.4 years, mean ± SD), which was significantly earlier than that of patients with LHON-A and LHON- patients (Fig 1A, S1 Table). All 26 patients carrying *OPA1* mutations insidiously presented with simultaneous binocular vision loss. The visual acuity of patients with *OPA1* mutations ranged from 0.01 to 0.6 (0.22 ± 0.16, mean ± SD), which was significantly higher than that of patients with LHON-A and LHON-SP (Fig 1B). The patients with missense mutations of *OPA1* had worse visual acuity (0.12 ± 0.03, mean ± SD) than those with other mutational types (0.23 ± 0.11, mean ± SD, P < 0.05) in *OPA1* mutations. The fundus examinations revealed symmetrical bilateral optic pallor in 23 patients (Fig 2G) and diffuse optic pallor (Fig 2H) in three patients. OCT showed that the RNFL thickness of patients with *OPA1* mutations was significantly reduced in all four quadrants (temporal, 38.88 ± 19.37 μm, P < 0.05; inferior, 86.625 ± 40.04 μm, P < 0.05; superior, 88.71 ± 32.39 μm, P < 0.05; and nasal, 54.79 ± 20.31 μm, P < 0.001) compared to age-matched controls. The VEP pattern of patients harbouring *OPA1* mutations reflected mild, moderate or serious damage.

**Table 2:** Summary of the mutations in *OPA1* and *OPA3*.

Gene	Exon	Patient ID	Nucleotide Change	Amino acid Change	State	Computed prediction		MAF	Reported
						PPH2/BDGP	SIF	1000G/EVS	
*OPA1*	4	Le2934	c. [449–34_44]insA; [=]	NA	NA	NA	NA	NA/NA	This study
	8	QT1470	c. [869G>A]; [=]	p. [R290Q]; [=]	Hete	D	D	NA/NA	[38]
	8	Le2424	c. [869G>A]; [=]	p. [R290Q]; [=]	Hete	D	D	NA/NA	[38]
	9	QT1325	c. [893G>A]; [=]	p. [S298N]; [=]	Hete	D	T	NA/NA	[38]
	12	Le2643	c. [1184T>G]; [=]	p. [V395G]; [=]	Hete	D	D	NA/NA	This study
	12	QT1451	c. [1187T>C]; [=]	p. [L396P]; [=]	Hete	D	T	NA/NA	[39,5]
	12	Le2174	c. [1187T>G]; [=]	p. [L396R]; [=]	Hete	PrD	D	NA/NA	[5]
	12	QT1319	c. [1198C>A]; [=]	p. [P400T]; [=]	Hete	PrD	D	NA/NA	This study
	12	Le2458	c. [1202G>A]; [=]	p. [G401D]; [=]	Hete	PrD	D	NA/NA	[40]
	12	Le2532	c. [1212+1G>A]; [=]	NA	Hete	SSA	NA	NA/NA	[41]
	12	Le2973	c. [1141A>T]; [=]	p. [T381S]; [=]	Hete	D	D	NA/NA	This study
	14	Le2974	c. [1334G>A]; [=]	p. [R445H]; [=]	Hete	D	D	NA/NA	[42]
	14	QT1752	c. [1316G>T]; [=]	p. [G439 V]; [=]	Hete	D	D	NA/NA	[35]
	16	Le2379	c. [1570_1571] insT; [=]	p. [Q524 Lfs*38]; [=]	NA	D	D	NA/NA	This study
	17	Le2883	c. [1610_1621 del]; [=]	p. [H537_T541delinsP]; [=]	NA	D	D	NA/NA	This study
	17	QT1657	c. [1667T>A]; [=]	p. [V556E]; [=]	Hete	D	D	NA/NA	This study
	20	Le2637	c. [1886C>G]; [=]	p. [S629*]; [=]	Hete	NA	NA	NA/NA	This study
	20	Le2904	c. [2014-40G>C]; [=]	NA	Hete	NA	NA	NA/NA	This study
	21	Le2217	c. [2131C>T]; [=]	p. [R711*]; [=]	Hete	NA	NA	NA/NA	[41]
	24	Le2519	c. [2496+2T>C]; [=]	NA	Hete	SSA	NA	NA/NA	[39]
	26	LE2799	c. [2707G>A]; [=]	p. [V903I]; [=]	Hete	PrD	T	NA/NA	This study
	27	Le2779	c. [2708_2711del]; [=]	p. [V903Gfs*3]; [=]	NA	NA	NA	NA/NA	[41]
	27	Le2411	c. [2708_2711del]; [=]	p. [V903Gfs*4]; [=]	NA	NA	NA	NA/NA	[41]
	27	Le2198	c. [2818+1G>A]; [=]	NA	Hete	SSA	NA	NA/NA	This study
	27	QT1292	c. [2818+1G>T]; [=]	NA	Hete	SSA	NA	NA/NA	[43]
	28	Le2726	c. [2819-2A>C]; [=]	NA	Hete	SSA	NA	NA/NA	[44]
*OPA3*	1	Le2661	c. [123C>G]; [=]	p. [ILe41 Met]; [=]	Hete	D	N	0.0000577	[45]
	1	Le2961	c. [123C>G]; [=]	p. [ILe41 Met]; [=]	Hete	D	N	0.0000577	[45]
	2	QT1063	c. [301T>C]; [=]	p. [Tyr101His]; [=]	Hete	D	D	NA	This study
	3	Le2103	c. [487G>A]; [=]	p. [Leu163Phe]; [=]	Hete	D	D	NA	This study

Note, Hete: heterozygous; D: Damaging; PrD: probably damaging; SSA: splicing site abolished; T: Tolerant; NA: not available; MAF: minor allele frequency.

### OPA3 mutations and clinical characteristics

Two novel and one known *OPA3* mutations in four (4/304, 1.3%) probands were detected among the 189 patients without mutations at the three primary mtDNA sites or in *OPA1*. One known mutation c.123.C>G of exon1 in two probands and a novel mutation c.301T>C of exon2 in one probands were detected in the isoform b of *OPA3* encoding a 179 amino acid protein, resulting in an amino acid substitution of Isoleucine to Methionine at position 41 and Tyrosine to Histidine at position 101. In addition, another novel mutation c.487G>A of exon 3 in one probands was detected in an isoform a of *OPA3* encoding a 180 amino acid protein, resulting in an amino acid substitution of Leucine to Phenylalanine at position 163. All mutations are summarized in S1 Table. None of the mutations were present in the 96 normal controls.

## Discussion

In the present study, we systematically analyzed the common gene mutation spectrum of Chinese patients with suspected hereditary optic atrophy with an onset age of no more than 14 years. Twenty-three *OPA1* pathogenic gene mutations, including 11 novel and 12 known mutations, were detected in 26 probands were detected. Our results further extended the *OPA1* mutation spectrum. The 23 detected mutations of *OPA1* included ten (43.5%) missense, seven (30.4%) splicing site, three frameshift, two nonsense and one in-del mutations. *OPA1* is mapped to 3q28-q29 with 8 different variants generated by alternative splicing sites of exons 4, 4b and 5b [46]. It encodes a dynamic-related protein in the mitochondrial membrane, which includes the GTPase domain, dynamic central region, putative GTPase effector domain and C-terminal coiled-coil domain [47]. *OPA1* was confirmed to be a gene that affects mitochondrial morphology and participates in the energy metabolism and apoptosis of cells [48]. It is a highly polymorphic gene with more than 333 pathogenic variants known to cause disease, 97.3% (324/333) of which are related to optic neuropathy [HGMD^®^ Human Gene Mutation Database]. Among the 11 novel *OPA1* mutations in this cohort, 8 mutations converge on the important functional regions, including 4/11 on the GTPase domain (Exons 8–15) and 4/11 on the dynamin central region (Exons 16–24). The remaining three novel *OPA1* mutations are located at exon 4, exon 24 and exon 26. The most common mutational type of the novel *OPA1* mutations (9/11) in this study is nucleotide substitution, which results in six missense mutations and three truncation mutations (splicing mutations, deletion or insertion of protein and stop gain of protein translation). Most of the truncation *OPA1* gene mutations cause haplo-insufficiency with decreased expression of approximately half of the *OPA1* proteins [39,49]. As Barboni et al. reported, we also observed that patients with missense mutations of the *OPA1* gene had more severe vision loss than patients with other mutational types of *OPA1* [50]. Our results indicated that the missense mutations of the *OPA1* gene located in the catalytic GTPase domain are more likely to cause a severe clinical course of DOA through a dominant-negative effect [35].

Identification of the hotspots in *OPA1* is an efficient method for detecting its pathogenic mutations. According to previous studies, the hotspots of *OPA1* mutations are distributed in exons, 8, 10, and 27, which code for the GTP domain or putative GED domain [51,52,29,53]. However, we did not detect any *OPA1* mutations in exon 2 or exon 10 in the present study. Nevertheless, the most common mutations in *OPA1* were located in exon 12 (30.8%) and accounted for 26.9% (7/26) of the mutations, which suggested that exon 12 could be a high frequency region of *OPA1* mutations in this cohort. The reported *OPA1* gene mutations are highly assembled in the GTP and putative GED domains in the Human Gene Mutation Database (54.2%). In addition, in this cohort, the proportion of *OPA1* gene mutations at the GTPase (Exon 8–15) and putative GED (Exon 27–28) domains accounted for 17/26 mutations (65.4%). These results indicated that the exons located at the GTPase and GED domains should be recommended for sequencing in the screening of *OPA1* gene mutations in Chinese patients with disease onset in childhood.

LHON is widely considered a disorder of adult or juvenile age, in which only some children are affected. The mutational frequency (29.3%) of LHON in this cohort was slightly lower than that of Chinese patients with LHON and any age of onset (38–46%) [29,30]. Among a different cohort in this study, the frequency of the three primary mtDNA mutations (18.9%) in 201 patients with a disease onset age of no more than 10 years (Table 1), was higher than that reported in an Italian study (11.5%) [31]. Our results showed that the three primary mtDNA mutations of LHON were the main cause of disease in Chinese patients with suspected childhood-onset hereditary optic neuropathy. In this study, we found that the G11778A mutational frequency among patients with disease onset earlier than 14 years (85.4%) was consistent with a former report of Chinese patients with onset of any age (G11778A, 90.2%); however, the frequencies of the T14484C (10.1%) and G3460A (4.5%) mutations were higher in this study than in a previous study (T14484C, 3.3%; G3460A, 0.4%) [30,29]. All three mtDNA mutations are located in genes encoding the subunits of mitochondrial complex I (CI), which is a member of the respiratory chain. Respiration rate and complex I activity were decreased in cybrids harbouring 11778G>A, 14484T>C or 3460G>A [54]. The visual deficits in individuals affected by these forms of hereditary optic atrophies involving mutations in either mtDNA, as in LHON, or in nuclear genes (*OPA1* and *OPA3*) encoding for mitochondrial membrane proteins mitochondrial membrane proteins [55,56,48]. This evidence indicates that altered mitochondrial function plays an essential role in the pathogenesis of optic neuropathies.

Clinically, discriminating between the genotype-phenotype characteristics of between LHON and DOA could help guide patients according to a specific genetic diagnosis. Both disorders specifically damage retinal ganglion cells and their axons by affecting the function of mitochondria and inducing apoptosis [57,4]. These phenotypes are also easily confused and difficult to distinguish from one another in the clinic, especially in young children. However, the disease processes of LHON and DOA are quite different: patients with LHON typically exhibit acute or subacute loss of vision due to synchronized cellular death, whereas patients with DOA exhibit slow, progressive loss of vision due to piecemeal loss of RGCs [4]. In the LHON group, we observed that 54 (60.7%) patients typically presented with typical circumpapillary telangiectasia of the vessels and edema of the nerve fiber layer (NFL) surrounding the optic disc (pseudoedema) in at least one eye [58]. In contrast, all 26 patients with *OPA1* mutations presented with symmetrical optic nerve pallor.

In the subgroup with an onset age earlier than 6 years, the mutational frequency of *OPA1* was 22.3% in our study, whereas only 6.4% of patients in this age range with LHON harboured mtDNA mutations. This finding suggests that among Chinese patients with suspected hereditary optic neuropathy, preschool children are mainly affected by *OPA1* mutations, but teenage children (7–14 years old) are primarily affected by mtDNA mutations. The disease was discovered in most of the patients with *OPA1* mutations (24/26) by their cautious parents or during routine health evaluations, which indicated that the age of DOA onset may be earlier than observed in the present report (4–6 years old) [6,32]. However, most (54/89) of the LHON patients or their guardians knew the specific timing of visual decline. Unexpectedly, we observed that 73% (19/26) of patients with *OPA1* mutations in the DOA group were initially misdiagnosed with LHON before genetic analysis, which indicated that DOA is not widely recognized as a major cause of hereditary optic neuropathy by ophthalmologists in China. Based on the results of the present study, we suggest that a valuable, effective detection strategy should be formulated for patients with suspected hereditary optic neuropathy. (1) In familial probands, the selection of *OPA1* or primary LHON-related mtDNA mutation sites for screening depends on the pedigree, which can reveal autosomal dominant inheritance or mtDNA transmission of the disorder. (2) In sporadic probands, when patients exhibit disease onset before preschool age, *OPA1* should be preferentially screened first, followed by analysis of the primary LHON-related mtDNA mutation sites. If patients with onset after preschool age exhibit optic nerve edema or tortuous vessels as well as optic nerve atrophy with thickening of the nasal and superior quadrants of the RNFL, then the primary LHON-related mtDNA mutation sites should be screened first, followed analysis of *OPA1*. (3) When neither *OPA1* nor primary LHON-related mtDNA mutations were found in these patients, the coding regions of *OPA3* were subsequently sequenced.

In conclusion, twenty-three *OPA1* mutations, including 11 novel and 12 known mutations, were detected in 26 probands in this study, and these results further extended the *OPA1* mutation spectrum. The three primary LHON-related mtDNA mutation sites were major cause of hereditary optic neuropathy in Chinese patients with suspected childhood disease onset. However, in affected preschool children, the mutational frequency of *OPA1* was higher than that of LHON-related mtDNA sites. These findings suggest that nuclear genes rather than the primary LHON-related mtDNA mutation sites should be preferentially screened in early-onset patients.

## Supporting Information

S1 Fig Pedigree and Sequence chromatography. Eleven potential pathogenic *OPA1* mutations in 11 probands and three *OPA3* mutations in four probands. The patients IDs and mutations are located above the mutant sequence chromatography. M: patients with mutant alleles; +: wild type allele.
(TIF)

S1 Table The clinical data of dominant optic atrophy patients with *OPA1* and *OPA3* mutations. (XLSX)

S2 Table Primers used for amplification and sequencing of *OPA1* in sanger sequencing. (XLSX)

S3 Table Primers used for amplification and sequencing of *OPA3* in sanger sequencing. (XLSX)
