# Supplementary figures and images for: Genetic and Clinical Analyses of DOA and LHON in 304 Chinese Patients with Suspected Childhood-Onset Hereditary Optic Neuropathy
Source: PLoS One. 2017 Jan 12;12(1):e0170090. doi: 10.1371/journal.pone.0170090 (PMC5230780; doi:10.1371/journal.pone.0170090)

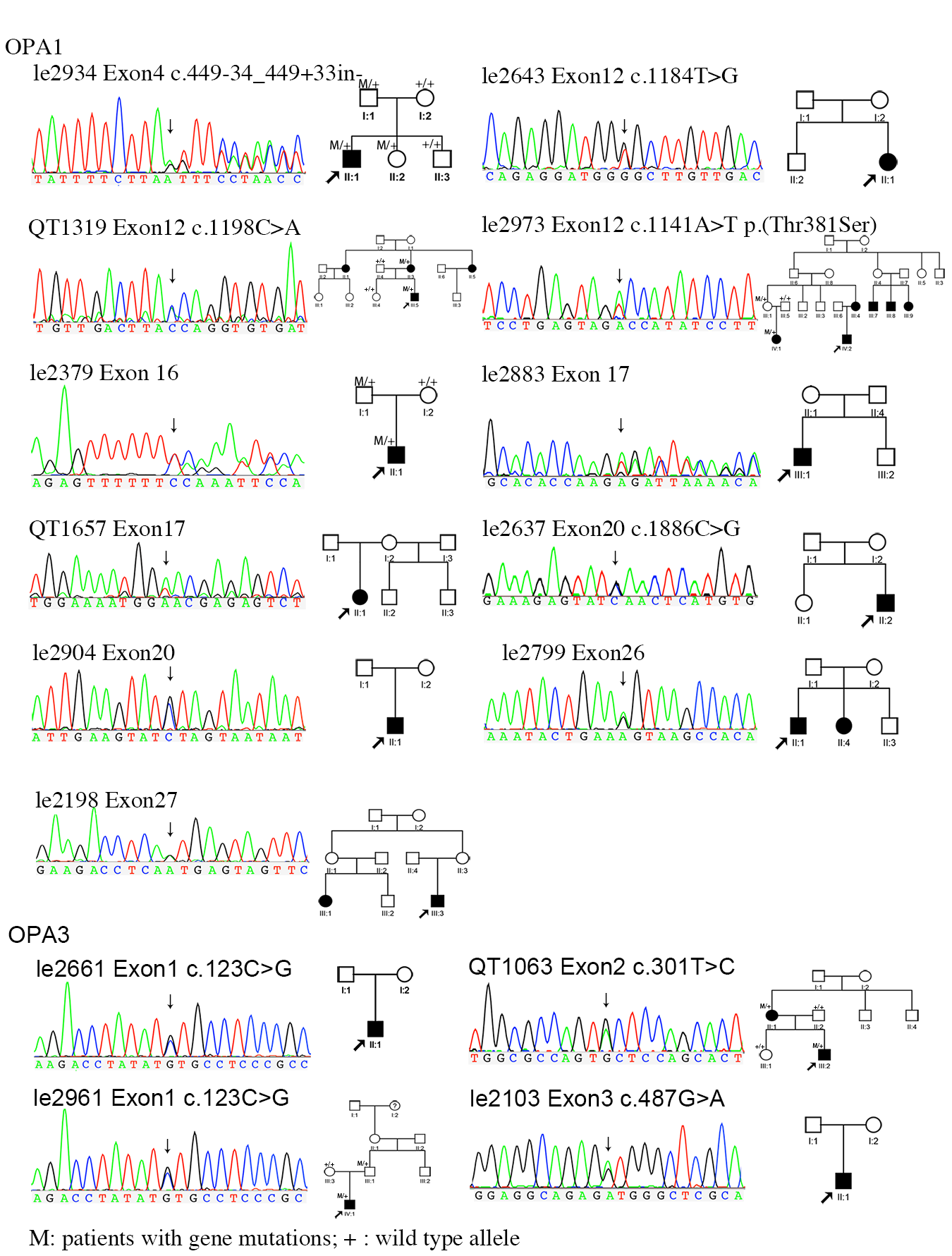

Supplement: S1 Fig — Eleven potential pathogenic OPA1 mutations in 11 probands and three OPA3 mutations in four probands. The patients IDs and mutations are located above the mutant sequence chromatography. M: patients with mutant alleles; +: wild type allele. (TIF) [file pone.0170090.s001.tif]
